# Trajectories of maternal depressive symptoms during pregnancy and the first 12 months postpartum and child externalizing and internalizing behavior at three years

**DOI:** 10.1371/journal.pone.0195365

**Published:** 2018-04-13

**Authors:** Dawn Kingston, Heather Kehler, Marie-Paule Austin, Muhammad Kashif Mughal, Abdul Wajid, Lydia Vermeyden, Karen Benzies, Stephanie Brown, Scott Stuart, Rebecca Giallo

**Affiliations:** 1 Faculty of Nursing, University of Calgary, Calgary, Alberta, Canada; 2 St John of God Health Care, Burwood, Australia; 3 University of New South Wales, Sydney, Australia; 4 Murdoch Childrens Research Institute, Parkville, Victoria, Australia; 5 Department of Obstetrics and Gynecology, University of Iowa, Iowa City, Iowa, United States of Amreica; Chiba Daigaku, JAPAN

## Abstract

**Background:**

Most evidence of the association between maternal depression and children’s development is limited by being cross-sectional. To date, few studies have modelled trajectories of maternal depressive symptoms from pregnancy through the early postpartum years and examined their association with social emotional and behavior functioning in preschool children. The objectives of this study were to: 1) identify distinct groups of women defined by their trajectories of depressive symptoms across four time points from mid-pregnancy to one year postpartum; and 2) examine the associations between these trajectories and child internalizing and externalizing behaviors.

**Methods:**

We analyzed data from the All Our Families (AOF) study, a large, population based pregnancy cohort of mother-child dyads in Alberta, Canada. The AOF study is an ongoing pregnancy cohort study designed to investigate relationships between the prenatal and early life period and outcomes for children and mothers. Maternal depressive symptoms were assessed using the Edinburgh Postnatal Depression Scale. Children’s behavioral functioning at age 3 was assessed using the Behavior Scales developed for the Canadian National Longitudinal Survey of Children and Youth. Longitudinal latent class analysis was conducted to identify trajectories of women’s depressive symptoms across four time points from pregnancy to 1 year postpartum. We used multivariable logistic regression to assess the relationship between trajectories of maternal depressive symptoms and children’s behavior, while adjusting for other significant maternal, child and psychosocial factors.

**Results:**

1983 participants met eligibility criteria. We identified four distinct trajectories of maternal depressive symptoms: low level (64.7%); early postpartum (10.9%); subclinical (18.8%); and persistent high (5.6%). In multivariable models, the proportion of children with elevated behavior symptoms was highest for children whose mothers had persistent high depressive symptoms, followed by mothers with moderate symptoms (early postpartum and subclinical trajectories) and lowest for minimal symptoms. After accounting for demographic, child and psychosocial factors, the relationships between depression trajectories and child hyperactivity/inattention, physical aggression (subclinical trajectory only) and separation anxiety symptoms remained significant.

**Conclusion:**

These findings suggest both externalizing and internalizing children’s behaviors are associated with prolonged maternal depressive symptoms. There is a good case for the need to move beyond overly simplistic clinical cutoff approaches of depressed/not depressed in screening for perinatal depression. Women with elevated depressive symptoms at clinical *and* subclinical levels need to be identified, provided with evidence-based treatment, and monitored with repeat screening to improve maternal mental health outcomes and reduce the risk of associated negative outcomes on children’s early social-emotional and behavior development.

## Introduction

Depressive symptoms during pregnancy and the transition to becoming a new parent is common, and can have adverse consequences for mother’s wellbeing, child development and family functioning. Estimates of the point prevalence of depressive symptoms ranges from 7% to over 20% during each trimester of pregnancy and postpartum month in the first year [1, 2]. Studies that have examined the course of maternal depressive symptoms during the perinatal and early postpartum period have consistently found that a history of depression and depression during pregnancy are significant risk factors for postpartum depression, suggesting that maternal depressive symptoms are often chronic or reoccur [3–5].

It is well established that maternal depression during pregnancy and the early postpartum period negatively impacts children’s social, emotional and behavioral development [6–9]. Except for a few studies [10], this evidence has primarily been generated from studies that are cross-sectional, focusing on the presence or absence of depressive symptoms at a hypothesized “critical period” such as pregnancy or the early postpartum. With the understanding that depressive symptoms during the perinatal period continue or recur for 40% to 80% of women [11, 12], more recent studies have begun to use longitudinal analysis methods to capture the chronicity and severity of maternal depressive symptoms and the associated impact on child development in young children. Indeed, in their classic review, Goodman and Gotlib (1999) highlight the need to understand the impact of recurring and persistent maternal depression over the course of time on a range of child developmental stages [13]. However, to date, only a few studies have modelled trajectories of maternal depressive symptoms from pregnancy through the early postpartum years and examined their association with child social emotional and behavior functioning in preschool aged children [11, 14, 15].

In a large population-based cohort study (n = 4167) in the Netherlands, Cents et al. (2013) modelled trajectories of maternal depressive symptoms from mid-pregnancy to three years postpartum [14]. They identified four maternal depressive symptoms trajectories: 1. no symptoms; 2. low symptoms; 3. moderate symptoms; and 4. high symptoms. Children of mothers assigned to trajectories with more severe depressive symptoms had more internalizing and externalizing behavior problems at age three than children with mothers assigned to the no symptom trajectory [14]. Giallo et al. (2015) undertook an analysis of data collected as part of the Maternal Health Study in Australia (n = 1085) [11]. Using latent class analysis, three trajectories of maternal depressive symptoms from pregnancy to four years postpartum were identified: 1) minimal symptoms; 2) subclinical symptoms; and 3) increasing and persistent high symptoms. Children with mothers assigned to the subclinical and high symptom trajectories had more emotional-behavioral difficulties at four years than children whose mothers were assigned to the minimal symptoms trajectory [11]. Similarly, Park et al. (2018) found three depression trajectories of low, increasing and decreasing from pregnancy to 3 years postpartum in a small study of Canadian mothers (N = 147)[16]. This study found that, compared with mothers with low symptoms, children of mothers with increasing depression symptoms had greater odds of problem behavior and those whose mothers experienced declining symptoms had comparable risks to those with consistently low symptoms. Finally, Van der Waerden et al. (2015) analyzed data from the EDEN mother-child cohort study, a large birth cohort in France (n = 1183) [15]. Using group-based semi-parametric modelling, five trajectories of maternal depressive symptoms spanning pregnancy to five years postpartum were identified:1) no symptoms; 2) persistent intermediate-level symptoms; 3) persistent high symptoms; 4) high symptoms in pregnancy only; and 5) high symptoms in the preschool period only. Compared with children whose mothers were assigned to the “no symptoms” trajectory, those whose mothers were in the “persistent high” or “persistent intermediate” trajectories had increased levels of emotional and behavioral difficulties at age five [15].

While the number of depression trajectories identified varies from three to five, these studies are consistent in reporting the presence of at least one low or minimal symptom trajectory, at least one moderate or sub-clinical symptoms trajectory and one high symptoms trajectory. These findings highlight the heterogeneous nature of maternal depressive symptoms across pregnancy and the early parenting years, challenging current thinking that categorizes women as “depressed” and “non-depressed.” Further, these studies have begun to build the evidence that children of mothers with a “sub-clinical symptoms” or “high symptoms” trajectory have more behavior problems than children with mothers who experience a “low depressive symptoms” trajectory.

The current study was undertaken to add to this limited body of literature by analyzing data from the All Our Families (AOF) study, a large, population based pregnancy cohort of mother and child dyads in Calgary, Alberta Canada [17]. The objectives of the current study were 1) to identify distinct groups of women defined by their trajectories of depressive symptoms across four time points from mid-pregnancy to one year postpartum and 2) to examine the associations between trajectories of maternal depressive symptoms and child behavior problems at age three on four outcomes: 1) hyperactivity and inattention; 2) physical aggression; 3) emotional and anxiety symptoms; and 4) separation anxiety, while accounting for socio-demographic, life history, pregnancy, early postpartum and concurrent factors.

## Method

### Study design

The All Our Families study (AOF), formerly the All Our Babies study, is an ongoing prospective pregnancy cohort study in Calgary, Canada that was established in 2008. AOF was developed to investigate the relationships between the prenatal and early life period and outcomes for infants, children and mothers. Recruitment and data collection methods have previously been described [17–19]. Briefly, women were recruited during pregnancy and asked to complete three questionnaires spanning pregnancy to four months postpartum and to consent to provide the research team with access to their obstetrical and birth records. Women who consented to be contacted for future research were asked to participate in subsequent follow-up questionnaires when their child was one, two and three years old. The initial sample for the AOF cohort was obtained through a population based multi-method recruitment strategy, involving primary health care offices, the public health laboratory service (Calgary Laboratory Service) and posters displayed in the community in places frequented by pregnant women. Eligibility criteria for the study included being less than 25 weeks’ gestation at the time of recruitment, at least 18 years of age, accessing prenatal care in Calgary, Canada, and proficiency in English to complete the written questionnaires. Recruitment and baseline data were collected between May 2008 and May 2011. The overall study was approved by the Conjoint Health Research Ethics Board at the University of Calgary. This analysis was approved by the Health Research Ethics Board at the University of Alberta (Pro00050020).

### Measures

Participants completed six questionnaires at the following time points: 1) before 25 weeks of pregnancy; 2) between 34 and 36 weeks of pregnancy; 3) at four months postpartum; 4) at one year postpartum; 5) at two years postpartum; and 6) at three years postpartum. These comprehensive questionnaires asked about socio-demographics, pregnancy, health service utilization, maternal mental and psychosocial health, obstetric and birth outcomes, child health, child development, and parenting. The questionnaires include both standardized scales and investigator-derived questions created specifically for the study when standardized measures were not available.

### Depressive symptoms

Depressive symptoms during pregnancy and the first 12 months postpartum were assessed using the Edinburgh Postnatal Depression Scale [20]. The EPDS is a widely used self-report instrument designed to measure symptoms of depression during the postpartum period. The EPDS has also been validated for use in pregnant populations [21, 22]. The EPDS consists of ten items rated on a 4-point scale based on “how you have felt in the past seven days, not just how you feel today.” Responses are scored from 0–3, for a possible range of scores of 0–30 with higher scores representing more depression. A score of greater than or equal to 13 has been recommended for identifying women with symptoms of major depression [20]. In the current study, the EPDS was included in each questionnaire at four time points: 1) at <25 weeks’ gestation; 2) 34–36 weeks’ gestation; 3) four months postpartum; and 4) one year postpartum. Internal consistency in the current sample as assessed by Cronbach’s alpha ranged from 0.85–0.87 across the four time points.

### Children’s externalizing and internalizing behaviors

Children’s behavioral functioning at three years of age was assessed using the Behavior Scales developed for the Canadian National Longitudinal Survey of Children and Youth (NLSCY) for two to three year old children [23]. Items for the NLSCY Behavior Scales were based on items from the Child Behavior Checklist (CBCL)[24]. The NLSCY Behavior Scales contain 25 items and comprise four subscales, including two externalizing subscales (hyperactivity and inattention; physical aggression) and two internalizing subscales (emotional disorder and anxiety; separation anxiety). Responses for all items are scored on a 3-point Likert scale of not true to very true or often true. A total score for each subscale is derived with higher scores indicating more behavioral problems. The possible range of scores for each subscale is 0–12 for the hyperactivity and inattention subscale, 0–16 for the physical aggression subscale, 0–12 for the emotional disorder and anxiety subscale and 0–10 for the separation anxiety subscale. A high symptoms category for each subscale was created based on scoring greater than or equal to one standard deviation (SD) above the mean of the AOF data. This corresponded to a cutoff score of 6 for the hyperactivity and inattention subscale, 8 for the physical aggression subscale, 3 for the emotional disorder and anxiety subscale and 4 for the separation anxiety subscale. Cronbach’s alphas for the four subscales for the current sample were 0.80 for the hyperactivity/inattention subscale, 0.76 for the physical aggression subscale, 0.60 for the emotional/anxiety disorder subscale and 0.58 for the separation anxiety subscale.

### Covariates

Information on a broad range of factors collected during pregnancy, the early postpartum and at three years postpartum were considered as covariates in the current study. The majority were single item questions unless a standardized scale is noted. Socio-demographic factors and mental health history (yes or no; measured during pregnancy at study intake) included maternal age (<25 years vs. ≥25 years), maternal education level (some high school/some post-secondary vs. completed post-secondary), family income (<$40,000 vs. $40,000–$79,999 vs. ≥$80,000), maternal primary language spoken in the home (English vs. other), parity (primiparous vs. multiparous)[16, 25, 26].

During pregnancy, anxiety symptoms during pregnancy were measured by the Spielberger State Anxiety Inventory (SSAI) [27]. A score of 40 or greater was used to classify women with symptoms of anxiety disorder in accordance with the authors’ scoring instructions. Early postpartum factors included gestational age at birth (<37 weeks vs. ≥37 weeks), child sex (male vs. female) and postpartum anxiety (SSAI <40 vs. SSAI ≥40). Concurrent factors, measured at three years postpartum, included maternal depression, anxiety and relationship happiness (happy vs. unhappy vs un-partnered). Depressive symptoms at 3 years postpartum were assessed using the Centre for Epidemiologic Studies Depression Scale (CES-D) [28]. The CES-D was designed to measure depressive symptoms in the general population. The CES-D consists of 20 items rated on a 4-point scale asking respondents “how often you have felt this way during the past week”. The CES-D has a possible range of scores of 0–60 with higher scores indicating more depressive symptoms. A score of 16 or greater is recommended for identifying depressive symptoms indicative of clinical depression. Anxiety symptoms were also assessed using the Spielberger State Anxiety Inventory (SSAI) at 3-years [27]. Concurrent depression (CES-D) and anxiety (SSAI) were measured at the same time point as the NLSCY behavior scales.

### Data analysis

Descriptive statistics were used to describe the demographic characteristics of all women who participated in the AOF three-year follow-up study. The mean, standard deviation and range were calculated for the main independent variable, maternal depressive symptoms on the EPDS (four time points) and the dependent variable, child behavior outcomes on the NLSCY Behavior Scales. Frequency and proportions were calculated to describe the number of women with depression (EPDS ≥13) at each time point and the number and proportion of children with elevated hyperactivity/inattention, physical aggression, emotional/anxiety disorder and separation anxiety scores (NLSCY Behavior Scale ≥1 SD above the mean) at three years of age.

Longitudinal latent class analysis was conducted to identify trajectories of women’s depressive symptoms (using continuous scores) across four time points (<25 weeks’ gestation, 34–36 weeks’ gestation, four months postpartum and one year postpartum) using MPlus version 7.11 [29]. A 1-class model was fit first followed by fitting successive models with increasing numbers of classes to identify the most parsimonious models with the fewest number of classes. Model solutions were evaluated by comparing Likelihood ratio statistic (L^2^), Akaike information Criterion (AIC) and Bayesian Information Criterion (BIC) across the successive models. Better fitting models have lower L^2^, AIC and BIC values. Additional model fit indices used to evaluate the model solutions included entropy, an index for assessing the precision of assigning latent class membership, and the Vuong-Lo-Mendall-Rubin Likelihood Ratio Test to test for significant differences between the models. Class size was also used as a criteria for model selection. The class membership of all participants in the study sample was saved and used in subsequent analyses.

A bivariate logistic regression analysis was used to measure the relationship between child behaviors (hyperactivity/inattention, physical aggression, emotional/anxiety symptoms and separation anxiety) and trajectories of maternal depression and covariates. Bivariate logistic regression results are presented as odds ratios (ORs) and 95% confidence intervals (CIs). Factors identified at the bivariate level based on statistical significance at the p<0.10 level, were considered for inclusion in the subsequent multivariable models. Multivariable logistic regression modeling was conducted to assess the relationship between trajectories of maternal depressive symptoms and children’s behavior while adjusting for other significant maternal, child and psychosocial factors. An alpha level of 0.05 or less was considered statistically significant for inclusion in the final logistic regression models. Multivariable logistic regression results are presented as adjusted odds ratios (AORs) and 95% confidence intervals (CIs).

Missing data were replaced using full information maximum likelihood in MPlus version 7.11 for the latent class modeling [29] and multiple imputation in Stata version 13.1 [30] for all variables used in the logistic regression analyses. Ten complete datasets were imputed using multiple imputation with chained equations (MICE). The logistic regression analyses were conducted using 1) the total sample with imputed data and 2) only cases with complete data. Given that the analyses yielded similar results, only those using imputed data are presented here.

## Results

### Sample characteristics

The current study was further restricted to the AOF participants with singletons only (due to unreliability of child outcome data for participants who had twins) and who participated in the three-year AOF follow-up questionnaire (1893/3316, 59.6%). The majority of women were between 25–34 years of age at recruitment, were married or in a common-law relationship, had post-secondary education, had family incomes ≥$80,000, were born in Canada and primarily spoke English at home (Table 1).

**Table 1:** Participant characteristics (n = 1983).

Characteristic	N (%)
Maternal age	
<25 years	133 (6.9%)
25–34 years	1416 (73.1%)
≥35 years	389 (20.1%)
Marital status	
Married/common law	1893 (95.6%)
Single, separated, divorced, widowed	87 (4.4%)
Highest education level	
Some high school/completed high school	162 (8.2%)
Some post-secondary/completed post-secondary	1492 (75.4%)
Some graduate school/completed graduate school	326 (16.5%)
Family income	
$39,999 or less	117 (6.1%)
$40,000–$79,999	405 (21.2%)
$80,000 or more	1392 (72.7%)
Country of birth	
Canada	1624 (81.9%)
Outside Canada	358 (18.1%)
Main language spoken at home	
English	1792 (90.4%)
Other	190 (9.6%)

Differences between demographic characteristics for women who completed the baseline AOF questionnaire and women who completed the three-year follow-up questionnaire have previously been described (Kingston et al., 2017, under review). Briefly, women who participated in the three-year follow-up questionnaire were more likely to be older, have a higher educational attainment, have family incomes of $80,000 or more, be born in Canada, and primarily speak English in their home than participants of the original AOF study (all p<0.05). In comparison, participants of the original study were also more likely to report depressive symptoms above the clinical cut-point (EPDS ≥13) during mid-pregnancy (baseline questionnaire) compared to participants of the 1-year follow-up questionnaire (8.1% vs. 6.4%, p = 0.022), with no significant differences observed at other time points.

### Descriptive statistics

Descriptive statistics for the depression variables and child behavior outcome variables are presented in Table 2. 1152 (58%) women had complete depression data from all 4 time points. The proportion of participants who reported EPDS scores ≥13 (indicative of symptoms of major depression) ranged from 4.7% to 6.4% across the four time points (Table 2). The proportion of children with high symptoms (≥1 SD above the mean) at three years of age was 16.9% for the hyperactivity/inattention subscale, 13.0% for the physical aggression subscale, 9.7% for the emotional/anxiety subscale and 18.9% for the separation anxiety subscale (Table 2). The extent of missing data across all the variables of interest in the analysis sample averaged 2.9%.

**Table 2:** Descriptive statistics for maternal depression and child externalizing and internalizing behavior variables.

Study variable	N	Mean	Standard Deviation	Median	Range	Cronbach’s α	Proportion at clinical cut-pointN (%)
Maternal depressive symptoms at <25 weeks of pregnancy (EPDS)	1975	4.98	4.25	4	0–22	0.85	126 (6.4%)
Maternal depressive symptoms at 34–36 weeks of pregnancy (EPDS)	1961	4.85	4.27	4	0–24	0.85	123 (6.3%)
Maternal depressive symptoms at 4 months postpartum (EPDS)	1934	4.17	4.23	3	0–30	0.86	91 (4.7%)
Maternal depressive symptoms at 1 year postpartum (EPDS)	1201	4.35	4.25	3	0–23	0.87	73 (6.1%)
Child hyperactivity and inattention symptoms at age 3 (NLSCY Behaviour Scales)	1976	3.17	2.33	3	0–12	0.80	333 (16.9%)
Child physical aggression symptoms at age 3 (NLSCY Behaviour Scales)	1970	4.47	2.75	4	0–16	0.76	256 (13.0%)
Child emotional/anxiety disorder symptoms at age 3 (NLSCY Behaviour Scales)	1980	0.86	1.25	0	0–10	0.60	191 (9.7%)
Child separation anxiety symptoms at age 3 (NLSCY Behaviour Scales)	1980	2.02	1.75	2	0–10	0.58	375 (18.9%)

EPDS Edinburgh Postnatal Depression Scale, NLSCY National Longitudinal Survey of Children and Youth.

### Trajectories of maternal depressive symptoms across pregnancy and the first postpartum year

Latent class models specifying 1–6 models were estimated (Table 3). The 4-class model was accepted as the final model after examining all indexes and tests of model fit. The 4-class model fit indexes (L^2^, AIC, BIC) were lower than the 1-, 2- and 3-class models indicating an improved fit of the data over the 1-, 2- and 3-class models. The Vuong-Lo-Mendell-Rubin likelihood ratio test indicated a significant difference between the 3- and 4-class models, suggesting that the 4-class model gives significant improvement in fit over the 3-class model. The 5-class model was not selected as the difference between the 4- and 5-class models was not significant. The entropy value for the 4-class model was high (0.83) suggesting acceptable precision in assigning individual cases to their appropriate class. Further an examination of the sample sizes of each latent class in the 4-class model found that all classes had a sufficient sample size with the smallest class (n = 112) constituting a meaningful class.

**Table 3:** Model fit indices for latent classes of depressive symptoms from pregnancy to one year postpartum.

Model	L^2^	BIC	AIC	Entropy	Vuong-Lo-Mendell-Rubin	p-value
1-class	-20262.02	40584.77	40540.03	-	-	-
2-class	-19219.41	38537.51	38464.81	0.849	1 vs. 2 classes	<0.001
3-class	-18998.59	38133.84	38033.17	0.816	2 vs. 3 classes	0.3476
4-class	-18882.17	37938.97	37810.35	0.827	3 vs. 4 classes	0.0349
5-class	-18782.20	37776.98	37620.39	0.803	4 vs. 5 classes	0.0560
6-class	-18717.49	37685.54	37500.99	0.790	5 vs. 6 classes	0.4458

L2 likelihood ratio statistic, BIC Bayesian information criterion, AIC Akaike information criterion, Vuong-Lo-Mendell-Rubin likelihood ratio test

**Fig 1:**
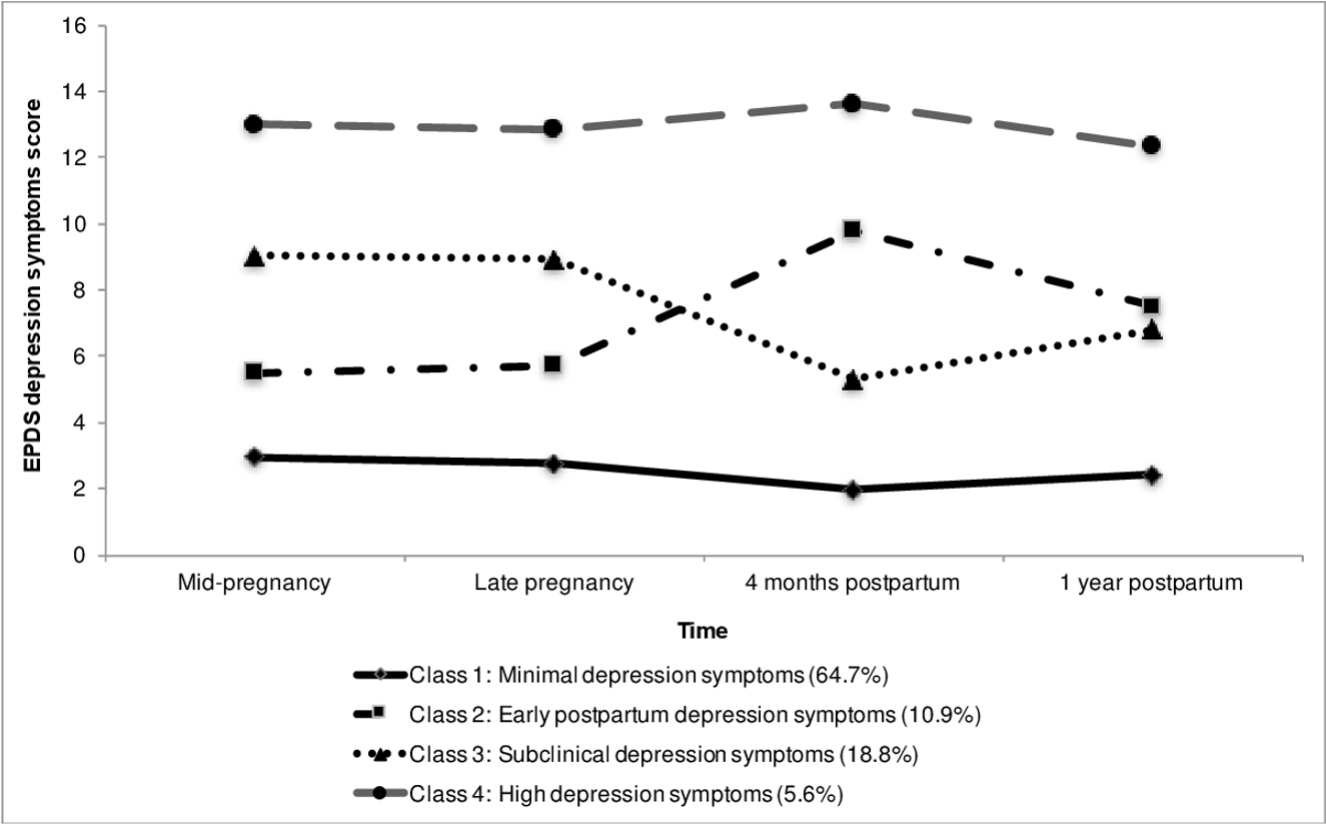
Estimated means of the EPDS for the four trajectories of maternal depressive symptoms from pregnancy to one year postpartum.

Fig 1 illustrates the four trajectory classes of maternal depressive symptoms. The first and largest trajectory consisted of women who reported “minimal depressive symptoms” from pregnancy to one year postpartum (n = 1283, 64.7%). The second trajectory, with a peak in the mean EPDS slightly higher than the sub-clinical group, consisted of women who reported “early postpartum depressive symptoms” (n = 216, 10.9%). The third trajectory consisted of women who reported “subclinical depressive symptoms” over time (n = 372, 18.8%). The fourth and smallest trajectory consisted of women who reported “high depressive symptoms” over time (n = 112, 5.6%).

### Trajectories of maternal depression and child internalizing and externalizing behaviors

Bivariate associations between maternal depression trajectories and child behavior outcomes (hyperactivity and inattention, physical aggression, emotional disorder and anxiety and separation anxiety) are presented in Table 4. Multivariable associations between maternal depression trajectories and children’s hyperactivity/inattention symptoms, physical aggression symptoms and other maternal, child and psychosocial characteristics are presented in Table 5.

**Table 4:** Bivariate relationships between maternal depression trajectories and child externalizing and internalizing behavior outcomes.

Maternal depression trajectories (EPDS)	Externalizing Behaviors		Internalizing Behaviors	
	High hyperactivity and inattention symptoms (n = 333) n (%)	High physical aggression symptoms (n = 256) n (%)	High emotional and anxiety disorder symptoms (n = 191) n (%)	High separation anxiety symptoms (n = 375) n (%)
Minimal symptoms	164 (12.8%)	120 (9.4%)	98 (7.7%)	187 (14.6%)
Early postpartum symptoms	55 (25.6%)	34 (15.9%)	30 (13.9%)	60 (27.8%)
Subclinical symptoms	78 (21.0%)	72 (19.5%)	44 (11.8%)	92 (24.7%)
High symptoms	36 (32.4%)	30 (26.8%)	19 (17.0%)	36 (32.1%)
Pearson’s Chi-square Test				
p-value	p<0.001	p<0.001	p<0.001	p<0.001

EPDS Edinburgh Postnatal Depression Scale

**Table 5:** Bivariate and final multivariable logistic regression models predicting children’s externalizing behaviors (hyperactivity and inattention and physical aggression) at age three.

	Hyperactivity and Inattention (NLSCY Behavior Scales)				Physical Aggression (NLSCY Behavior Scales)			
	Bivariate OR (95% CI)	P-value	Multivariable OR (95% CI)	P-value	Bivariate OR (95% CI)	P-value	Multivariable OR (95% CI)	P-value
**Maternal depression trajectories (EPDS)**								
Minimal symptoms	Reference		Reference		Reference		Reference	
Early postpartum symptoms	2.32 (1.64–3.28)	<0.001	1.84 (1.24–2.74)	0.002	1.79 (1.19–2.71)	0.005	1.22 (0.76–1.95)	0.402
Subclinical symptoms	1.80 (1.34–2.43)	<0.001	1.46 (1.04–2.06)	0.030	2.30 (1.68–3.17)	<0.001	1.64 (1.13–2.36)	0.009
High symptoms	3.30 (2.15–5.06)	<0.001	2.17 (1.22–3.88)	0.009	3.50 (2.21–5.54)	<0.001	1.61 (0.85–3.05)	0.144
**Maternal age**								
< 25 years	1.92 (1.29–2.87)	0.001	1.90 (1.21–2.97)	0.005	1.70 (1.09–2.66)	0.020	1.59 (0.96–2.62)	0.070
≥ 25 years	Reference		Reference		Reference		Reference	
**Maternal education**								
High school/some post-secondary	1.09 (0.82–1.45)	0.569	-		1.46 (1.08–1.97)	0.015	1.17 (0.84–1.64)	0.355
Graduated post-secondary	Reference		-		Reference		Reference	
**Family income**								
< $40,000	0.95 (0.56–1.61)	0.857	0.70 (0.39–1.25)	0.230	1.04 (0.59–1.82)	0.894	-	
≥ $40,000–$79,999	1.27 (0.96–1.68)	0.093	1.05 (0.77–1.42)	0.761	1.23 (0.90–1.68)	0.204	-	
≥ $80,000	Reference		Reference		Reference		-	
**Maternal primary language spoken at home**								
English	Reference		-		Reference		-	
Other	1.21 (0.83–1.78)	0.317	-		0.74 (0.45–1.22)	0.245	-	
**Parity**								
Primiparous	Reference		Reference		Reference		Reference	
Multiparous	0.77 (0.60–0.97)	0.029	0.78 (0.61–0.99)	0.044	1.70 (1.30–2.22)	<0.001	1.84 (1.38–2.44)	<0.001
**History of a mental health disorder(s)**								
Yes	1.81 (1.42–2.30)	<0.001	1.44 (1.11–1.88)	0.006	2.05 (1.57–2.67)	<0.001	1.65 (1.23–2.21)	0.001
No	Reference		Reference		Reference		Reference	
**Symptoms of anxiety during pregnancy** (SSAI)								
Yes (SSAI ≥40)	1.68 (1.24–2.28)	0.001	0.95 (0.64–1.40)	0.795	1.87 (1.35–2.59)	<0.001	0.83 (0.54–1.27)	0.389
No (SSAI <40)	Reference		Reference		Reference		Reference	
**Gestational age**								
≥37 weeks	Reference		-		Reference		-	
<37 weeks	0.82 (0.49–1.37)	0.452	-		0.68 (0.37–1.25)	0.215	-	
**Child sex**								
Female	Reference		Reference		Reference		Reference	
Male	1.25 (0.98–1.59)	0.077	1.27 (0.99–1.63)	0.058	1.37 (1.05–1.79)	0.022	1.43 (1.08–1.90)	0.013
**Symptoms of postpartum anxiety** (SSAI)								
Yes (SSAI ≥40)	1.94 (1.43–2.63)	<0.001	0.97 (0.65–1.45)	0.890	2.01 (1.45–2.79)	<0.001	0.95 (0.61–1.47)	0.813
No (SSAI <40)	Reference		Reference		Reference		Reference	
**Symptoms of depression at 3 years postpartum (CES-D)**								
Yes (CES-D ≥16)	2.50 (1.85–3.38)	<0.001	1.33 (0.90–1.97)	0.152	3.46 (2.53–4.74)	<0.001	2.08 (1.38–3.14)	0.001
No (CESD <16)	Reference		Reference		Reference		Reference	
**Symptoms of anxiety at 3 years postpartum (SSAI)**								
Yes (SSAI ≥40)	2.42 (1.82–3.21)	<0.001	1.58 (1.09–2.29)	0.015	2.82 (2.08–3.81)	<0.001	1.62 (1.08–2.43)	0.019
No (SSAI <40)	Reference		Reference		Reference		Reference	
**Happiness in partner relationship**								
Happy	Reference		Reference		Reference		Reference	
Unhappy	1.36 (0.94–1.97)	0.099	0.90 (0.60–1.34)	0.603	1.54 (1.04–2.29)	0.033	0.91 (0.59–1.41)	0.667
No partner	1.17 (0.65–2.11)	0.601	0.77 (0.41–1.45)	0.418	1.90 (1.07–3.36)	0.027	1.31 (0.70–2.44)	0.394

NLSCY National Longitudinal Survey of Children and Youth, EPDS Edinburgh Postnatal Depression Scale, SSAI Spielberger State Anxiety Inventory, CES-D Centre for Epidemiological Studies Depression Scale.

### Hyperactivity/Inattention symptoms

In the final multivariable model, having a mother assigned to the early postpartum (AOR 1.84, 95% CI 1.24–2.74), subclinical (AOR 1.46, 95% CI 1.04–2.06) or high (AOR 2.17, 95% CI 1.22–3.88) depressive symptoms trajectory continued to be associated with an increased risk of high hyperactivity and inattention symptoms at age three after adjusting for other factors (Table 5). Additional maternal factors associated with high hyperactivity and inattention symptoms included younger maternal age, being a first-time mother (primiparous), having a history of poor mental health and experiencing symptoms of anxiety when their child was three years old (Table 5).

### Physical aggression symptoms

The final multivariable model identified that having a mother assigned to the subclinical symptoms trajectory (AOR 1.64, 95% CI 1.13–2.36), but not early postpartum or high depressive symptoms trajectories, was associated with an increased risk of physical aggression symptoms at age three after adjusting for other factors. Additional factors retained in the multivariable model included being multiparous, having a history of poor mental health, having a male child, and experiencing symptoms of depression or anxiety when their child was three years old (Table 5).

### Trajectories of maternal depression and child internalizing behaviors

Bivariate and multivariable associations between children’s emotional/anxiety disorder symptoms, separation anxiety symptoms and maternal depression trajectories and other maternal, child and psychosocial characteristics are presented in Table 6.

**Table 6:** Bivariate and final multivariable logistic regression model predicting children’s Internalizing behaviors (emotional/anxiety disorder and separation anxiety) at age three.

	Emotional disorder and anxiety (NLSCY Behavior Scales)				Separation anxiety (NLSCY Behavior Scales)			
	Bivariate OR (95% CI)	P-value	Multivariable OR (95% CI)	P-value	Bivariate OR (95% CI)	P-value	Multivariable OR (95% CI)	P-value
**Maternal depression trajectories (EPDS)**								
Minimal symptoms	Reference		Reference		Reference		Reference	
Early postpartum symptoms	1.95 (1.26–3.02)	0.003	1.44 (0.87–2.38)	0.151	2.24 (1.60–3.13)	<0.001	2.16 (1.48–3.16)	<0.001
Subclinical symptoms	1.62 (1.11–2.36)	0.012	1.23 (0.80–1.91)	0.348	1.91 (1.44–2.54)	<0.001	1.50 (1.08–2.08)	0.014
High symptoms	2.47 (1.45–4.22)	0.001	1.40 (0.67–2.92)	0.367	2.76 (1.80–4.22)	<0.001	1.89 (1.07–3.34)	0.029
**Maternal age**								
< 25 years	1.00 (0.55–1.81)	0.997	-		1.40 (0.93–2.12)	0.107	-	
≥ 25 years	Reference				Reference		-	
**Maternal education**								
High school /some post-secondary	0.92 (0.63–1.34)	0.676	-		1.58 (1.22–2.05)	0.001	1.42 (1.07–1.89)	0.016
Graduated post-secondary	Reference		-		Reference		Reference	
**Family income**								
< $40,000	0.80 (0.40–1.61)	0.526	-		2.20 (1.46–3.34)	<0.001	1.38 (0.86–2.21)	0.179
≥ $40,000–$79,999	1.20 (0.84–1.71)	0.319	-		1.55 (1.19–2.03)	0.001	1.19 (0.90–1.59)	0.226
≥ $80,000	Reference				Reference		Reference	
**Maternal primary language spoken at home**								
English	Reference		Reference		Reference		Reference	
Other	1.81 (1.18–2.77)	0.006	1.97 (1.26–3.07)	0.003	2.36 (1.71–3.28)	<0.001	2.13 (1.49–3.04)	<0.001
**Parity**								
Primiparous	Reference		Reference		Reference		-	
Multiparous	0.59 (0.43–0.80)	0.001	0.57 (0.42–0.78)	0.001	0.94 (0.75–1.18)	0.621	-	
**History of a mental health disorder(s)**								
Yes	1.99 (1.47–2.68)	<0.001	1.76 (1.27–2.44)	0.001	1.35 (1.07–1.70)	0.011	1.09 (0.84–1.41)	0.515
No	Reference		Reference		Reference		Reference	
**Symptoms of anxiety during pregnancy (SSAI)**								
Yes (SSAI ≥40)	1.49 (1.02–2.20)	0.040	0.88 (0.54–1.45)	0.622	1.69 (1.27–2.26)	<0.001	1.08 (0.75–1.56)	0.686
No (SSAI <40)	Reference		Reference		Reference		Reference	
**Gestational age**								
≥37 weeks	Reference		-		Reference		-	
<37 weeks (preterm)	0.72 (0.36–1.44)	0.352	-		1.12 (0.72–1.76)	0.615	-	
**Child sex**								
Female	Reference		-		Reference		-	
Male	0.97 (0.72–1.31)	0.832	-		0.92 (0.73–1.15)	0.442	-	
**Symptoms of postpartum anxiety (SSAI)**								
Yes (SSAI ≥40)	1.76 (1.20–2.59)	0.004	1.07 (0.65–1.77)	0.798	1.51 (1.11–2.05)	0.009	0.74 (0.49–1.10)	0.137
No (SSAI <40)	Reference		Reference		Reference		Reference	
**Symptoms of depression at 3 years postpartum (CES-D)**								
Yes (CES-D ≥16)	2.35 (1.63–3.40)	<0.001	1.55 (0.96–2.49)	0.071	2.29 (1.70–3.08)	<0.001	1.51 (1.03–2.22)	0.033
No (CESD <16)	Reference		Reference		Reference		Reference	
Symptoms of anxiety at 3 years postpartum (SSAI)								
Yes (SSAI ≥40)	2.00 (1.40–2.86)	<0.001	1.22 (0.77–1.95)	0.401	1.77 (1.34–2.35)	<0.001	1.04 (0.72–1.51)	0.820
No (SSAI <40)	Reference		Reference		Reference		Reference	
**Happiness in partner relationship**								
Happy	Reference				Reference		Reference	
Unhappy	1.21 (0.75–1.94)	0.431	-		1.97 (1.41–2.75)	<0.001	1.55 (1.09–2.22)	0.016
No partner	1.11 (0.52–2.34)	0.791	-		2.04 (1.24–3.38)	0.005	1.48 (0.87–2.53)	0.152

NLSCY National Longitudinal Survey of Children and Youth, EPDS Edinburgh Postnatal Depression Scale, SSAI Spielberger State Anxiety Inventory, CES-D Centre for Epidemiological Studies Depression Scale.

### Emotional disorder and anxiety symptoms

At the bivariate level, compared to children with mothers assigned to the minimal depressive symptoms trajectory, children with mothers assigned to the early postpartum, subclinical and high depressive symptom trajectories were more likely to have high emotional/anxiety disorder symptoms (Table 6). Bivariate associations between children’s emotional/anxiety symptoms and other maternal and child factors are presented in Table 6.

The final multivariable model found that being assigned to the early postpartum, subclinical or high depressive symptoms trajectories was not associated with an increased risk of having a child with emotional/anxiety symptoms at age three after adjusting for other factors. Maternal risk factors that were associated with an increased risk of high emotional/anxiety disorder symptoms in the final multivariable model included speaking English as a second language, being a first-time mother (primiparous) and having a history of poor mental health (Table 6).

### Separation anxiety symptoms

At the bivariate level, children with mothers assigned to the early postpartum, subclinical and high symptoms trajectories were more likely to have high separation anxiety symptoms compared to children with mothers assigned to the minimal depressive symptoms trajectory (Table 6).

In final multivariable model, having a mother assigned to the early postpartum, subclinical or high depressive symptoms trajectories was associated with an increased risk of separation anxiety symptoms at age three after adjusting for other factors (Table 6). Additional risk factors for high separation anxiety symptoms identified in the final multivariable model included having lower educational attainment, speaking English as a second language, experiencing symptoms of depression at three years postpartum and reporting being unhappy in their partner relationship at three years postpartum (Table 6).

## Discussion

The current study identified four distinct trajectories of maternal depressive symptoms across the prenatal and first postpartum year: low level symptoms, early postpartum symptoms, subclinical symptoms and persistent high symptoms. For all four child behavior problems (hyperactivity/inattention, physical aggression, emotional/anxiety symptoms, separation anxiety) the proportion of children with elevated behavior symptoms was highest for children whose mothers had persistent high depressive symptoms, followed by mothers with moderate symptoms (early postpartum and subclinical trajectories) and lowest for minimal symptoms. After accounting for demographic, child and psychosocial factors, the relationships between depression trajectories and child hyperactivity/inattention, physical aggression (subclinical trajectory only) and separation anxiety symptoms were attenuated but remained significant in the final model, whereas emotional/anxiety symptoms were not significant.

The number of trajectories we report aligns with the two to six trajectories reported in a recent systematic review of 23 studies examining perinatal depressive symptom trajectories and symptom profiles [31]. As in the review, the low trajectory represented the largest trajectory group (65%) and the high symptoms trajectory represented the smallest trajectory group (6%), both whose depressive symptoms followed a stable pattern of low/high symptoms over time [31].

Clinically, women assigned to the high symptoms trajectory (mean EPDS 12–14) would likely be identified as having symptoms of major depression using the recommended screening cut-off of 13 or greater on the widely used EPDS. However, those assigned to both the early postpartum or the subclinical symptoms trajectory may not be identified as their mean scores (EPDS 5–10) are below the recommended clinical cut-off points. Overall, the substantial proportion of women in the sub-clinical and low symptom groups, and the significant associations of these degrees of symptoms with adverse child outcomes provide evidence of the need for early identification and support for women across pregnancy and the first postpartum year across all levels of symptom severity. Mental health screening that identifies various degrees of symptoms offers greater utility in using screening results to match women’s needs to the most appropriate level of care required–from self-management to professional support.

Children whose mothers experienced early postpartum, subclinical and high depressive symptoms were at an increased risk of hyperactivity/inattention and physical aggression symptoms. For both externalizing behavior outcomes, a dose response relationship was observed where the proportion of children with elevated behavior symptoms was highest for children whose mothers had high depressive symptoms, followed by mothers with moderate symptoms (early postpartum and subclinical trajectories) and lowest for minimal symptoms. These findings provide further support for an association between ongoing maternal depressive symptoms at both clinical and subclinical levels and the risk of behavior problems in preschool aged children [14, 15, 32]. After adjusting for socio-demographic, psychosocial and child factors associated with behavior problems, the early postpartum, subclinical and high depression trajectories were associated with an increased risk of hyperactivity/inattention symptoms, while only the subclinical trajectory remained a significant predictor of high physical aggression symptoms.

Like the externalizing behavior findings, the proportion of children experiencing internalizing problems (emotional/anxiety symptoms and separation anxiety symptoms) at age three was highest for children whose mothers had more severe depressive symptoms across the perinatal and early postpartum period. For both emotional/anxiety symptoms and separation anxiety symptoms, the proportion of children with elevated symptoms was highest for children whose mothers had persistent high symptoms, followed by early postpartum symptoms, subclinical symptoms, and lowest for minimal symptoms. After adjusting for socio-demographic, psychosocial and child factors, all three trajectories of elevated depressive symptoms: early postpartum, subclinical and high symptoms were associated with an increased risk of high separation anxiety symptoms. None of the maternal depression trajectories were significantly associated with high emotional/anxiety symptoms after adjusting for other maternal and child factors.

The differential effects that we found between varying degrees of depression and different child outcomes highlights the complexity that exists in understanding the roles of timing, duration, and severity on child health and development. Historically, these factors have not consistently been taken into account when examining maternal psychological distress and child outcomes. However, recent studies of maternal stress demonstrate that timing of stress (prenatal vs postnatal; early pregnancy vs late) has a clear effect on whether the child outcome is positive or negative, as does the type of distress [33–35]. Data from disaster studies have demonstrated that many of the prenatal effects (positive and negative) are transmitted to the child through epigenetic mechanisms that result in adaptive behaviors and physiological responses [36]. In her review of the impact of stress, anxiety and depression on child outcomes, Glover notes that an adverse prenatal environment (i.e., due to in utero depression) may epigenetically program the child to develop adaptive behaviours, such as aggression, that prepare him to survive in an adverse extrauterine world [37].

While examining the relationship between maternal depression trajectories and children’s behavior problems, the comprehensive data collection conducted during the AOF study allowed us to account for socio-demographic, child and psychosocial characteristics in pregnancy, the early postpartum and concurrent factors at three years. Additional mental health variables (history of poor mental health, depression and anxiety at three years) retained in the final models provide further support for the recurring and comorbid nature of mental health disorders [38–40]. Being a male child has been associated with an increased risk of externalizing behavior problems [41–43]. We found being a male child was associated with an increased risk of physical aggressive symptoms, but not hyperactivity and inattention problems or internalizing behavior outcomes.

Interestingly, parity had differential effects on child behavior outcomes. Being a first-time mother was associated with an increased risk of hyperactivity/inattention symptoms and emotional/anxiety symptoms in children at age three, while multiparity was associated with an increased risk of physical aggression symptoms after accounting for trajectories of maternal depression and adjusting for other factors in the final models. Being multiparous suggests that the study child is more likely to have siblings, which may provide more opportunities for physical aggressive play as has previously been reported [44]. Lower relationship satisfaction at three years postpartum was retained in the final model for child separation anxiety. This finding is in line with research that has reported associations between lower parental marital satisfaction and negative family environment and child anxiety problems [45–47]. Primarily speaking a language other than English was retained in the final models for both internalizing outcomes, but not externalizing outcomes. Ethnic minority has previously been linked to internalizing problems, but not externalizing problems in children and mothers who speak English as a second language are more likely to be of ethnic minority [48]. The remaining socio-demographic risk factors retained in the final models—lower maternal age and lower educational attainment–likely reflect lower socioeconomic status, which has previously been linked to increased child behavior problems [7].

There is a need to further understand how maternal depression and other key factors we have identified impact child development. Goodman and Gotlib note that risk may be conferred to the child through heritability of depression, dysfunctional neuroregulatory mechanisms, negative maternal cognitions and behaviors, and the “stressful context” of children’s lives, all of which may contribute to the child’s risk as their cognitions, emotions, and behaviors are affected [13]. As such, in addition to addressing maternal depression through screening and treatment, further amelioration of risk may be accomplished by targeting parenting approaches and the parent-child relationship.

### Strengths and limitations

Our study has several strengths. In most regards, the AOF study represents a large, population based sample of urban Canadian mothers and children [19]. It used a longitudinal study design with frequent follow-up from pregnancy through 1-year postpartum and the early childrearing years, including repeated assessments with consistent measures of maternal depression. In addition, the AOF study used validated measures of maternal mental health and child development, and included numerous maternal, family, and child characteristics potentially associated with children’s emotional and behavioral development.

Our results should be considered in the context of the following limitations. First, maternal depressive symptoms and child behavior symptoms were both based on maternal report using standardized symptom measures, not clinical diagnostic interviews. Mothers with poor mental health may be more likely to rate their child’s behavior negatively than mothers who don’t report mental health challenges. Ideally, to strengthen the findings, the outcome measure of child behavior would have been based on responses or observations from multiple informants (father, childcare provider, preschool teacher). Second, differences were found for women who participated in the 1-year follow-up questionnaire compared to women who completed the first AOF questionnaire. These differences reflect a lower risk sample with higher socioeconomic status that included a greater proportion of women who were older maternal age, had higher educational attainment, higher family income, were Canadian born and primarily English speaking. With the average Canadian family income at $78,700 (Statistics Canada) at the time of recruitment, women who participated in the 1-year follow-up questionnaire may not be representative of the Canadian population. Consequently, caution should be taken with generalizing the study findings to women with lower socioeconomic characteristics and new immigrants. Participants of the original study were more likely to report depressive symptoms above the clinical cut-point (EPDS ≥13) during mid-pregnancy (baseline questionnaire) compared to women retained in the 1-year follow-up. Thus, the proportion of women with depressive symptoms may be underestimated in the final sample.

## Conclusion

Women’s depressive symptoms from pregnancy through the first postpartum year followed four distinct trajectories defined by low level symptoms, early postpartum symptoms, subclinical symptoms and persistent high symptoms. For all four child behavior problems examined (hyperactivity/inattention, physical aggression, emotional/anxiety symptoms, separation anxiety), a dose response relationship was observed where the proportion of children with elevated behavior symptoms was highest for children whose mothers had persistent high depressive symptoms, followed by mothers with moderate symptoms (early postpartum and subclinical trajectories) and lowest for minimal symptoms. Even after accounting for demographic, child and psychosocial factors, the relationships between depression trajectories and child hyperactivity/inattention, physical aggression (subclinical trajectory only) and separation anxiety symptoms remained. These findings draw attention to the need to move beyond a “depressed/not depressed” approach to screening for depression in the perinatal period. Women with elevated depressive symptoms at clinical and subclinical levels need to be identified, monitored, and when necessary, provided with evidence based treatment to improve maternal mental health outcomes and reduce the risk of the associated negative outcomes on children’s early social-emotional and behavior development. Further study of women with subclinical symptoms is justified given they represent approximately 30% of mothers in our sample, their pattern of depressive symptoms was significantly different from the low symptoms group and they showed symptoms that persisted across the critical period from pregnancy to one year postpartum.
